# Measurement invariance properties and external construct validity of the short Warwick-Edinburgh mental wellbeing scale in a large national sample of secondary school students in Wales

**DOI:** 10.1186/s12955-019-1204-z

**Published:** 2019-08-14

**Authors:** G.J. Melendez-Torres, Gillian Hewitt, Britt Hallingberg, Rebecca Anthony, Stephan Collishaw, Jeremy Hall, Simon Murphy, Graham Moore

**Affiliations:** 1 Centre for the Development and Evaluation of Complex Interventions for Public Health Improvement (DECIPHer), School of Social Sciences, Cardiff University, 1-3 Museum Place, Cardiff, CF10 3BD UK; 2 Child and Adolescent Psychiatry Section, Institute of Psychological Medicine and Clinical Neurosciences, Cardiff University School of Medicine, Cardiff, UK; 3 Neuroscience and Mental Health Research Institute and the MRC Centre for Neuropsychiatric Genetics and Genomics, Cardiff University, Cardiff, UK

**Keywords:** Mental wellbeing, Adolescents, Measure, Psychometric properties

## Abstract

**Purpose:**

The study of mental wellbeing requires reliable, valid, and practical measurement tools. One of the most widely used measures of mental wellbeing is the Warwick-Edinburgh Mental Wellbeing Scale (WEMWBS). Our aim was to examine the psychometric properties of SWEMWBS (a brief seven-item version) in a ‘real-world’ population sample of young people.

**Methods:**

We used data from the 2017 School Health Research Network Student Health and Wellbeing Survey, completed by 103,971 students in years 7 to 11 from 193 secondary schools in Wales. We first estimated polychoric correlation matrices for the whole sample and by school year, and undertook a principal components analysis to check for configural invariance. Subsequently, we used a multiple-groups structural equation model with successively greater constraints to test measurement invariance. To examine external construct validity, we calculated correlations between the SWEMWBS score and four covariates: life satisfaction, somatisation, school pressure and bullying victimisation.

**Results:**

Parallel analysis suggested that extraction of one factor was appropriate both overall and in each year group. Inspection of standardised loadings suggested that four items had progressively stronger correlations with the factor as students are older, but change in fit indices between models suggested that loadings and thresholds, but not residual variances, were invariant by age group. SWEMWBS scores were moderately correlated with measures of life satisfaction and somatisation, and weakly to moderately correlated with school pressure and bullying victimisation.

**Conclusions:**

This study adds to the growing evidence that SWEMWBS is appropriate for measuring mental wellbeing in young people and suggests that SWEMWBS is appropriate for tracking the development of wellbeing across adolescence.

**Electronic supplementary material:**

The online version of this article (10.1186/s12955-019-1204-z) contains supplementary material, which is available to authorized users.

## Background

Mental wellbeing has emerged as an important construct in population health. In contrast to illness-defined perspectives on mental health, mental wellbeing is defined as ‘a state in which an individual can realize his or her own abilities, cope with the normal stresses of life, work productively and fruitfully, and is able to make a contribution to his or her community’ [1]. That is, mental wellbeing is to be promoted by health services, while mental illness is to be prevented or treated. Precise definitions of the dimensions of mental wellbeing are difficult to pin down, but a substantial literature on adult wellbeing suggests that mental wellbeing corresponds closely with psychological and functional wellbeing, including sub-constructs such as self-acceptance, positive relationships, autonomy and life purpose [2]. However, mental wellbeing can also draw from hedonic wellbeing, or aspects of wellbeing that relate to feelings and life satisfaction [3].

 The study of mental wellbeing in populations requires reliable, valid, and practical measurement tools. A particularly important question concerns the extent to which measures developed for use in adult populations can be utilised in child and adolescent samples. One of the most widely used measures of mental wellbeing is the Warwick-Edinburgh Mental Wellbeing Scale (WEMWBS), a 14-item questionnaire covering both psychological functioning and subjective wellbeing facets of mental wellbeing [4]. A brief seven-item version (SWEMWBS) was subsequently developed [5]. The authors stated that SWEMWBS had preferable psychometric properties to the full version, though it is focused more on functioning than subjective aspects of mental wellbeing. A recent study measuring well-being in a nationally-representative, population-based survey in Denmark found that both WEMWBS and SWEMWBS had high internal consistency and recommended the use of SWEMWBS in epidemiological research [6]. A brief version may also be of particular use in population research where practical constraints often restrict the scope for inclusion of detailed assessments in surveys, for example in research in schools. A validation of the 14-item WEMWBS in a sample of English and Scottish adolescents aged 13–16 found acceptable consistency, reliability and external construct validity [7], but thus far most studies examining the psychometric properties of SWEMWBS have been undertaken in adults [5, 8, 9].

Mental wellbeing has become a focus of health promotion interventions in school settings [10], as well as a target of education and health policy [11]. Adolescence is a developmental period characterised by rapid developmental change. Whilst there are well-documented developmental changes in mental health problems such as depression across childhood and adolescence [12], less is known about development of mental wellbeing. Providing estimates of age differences in mental wellbeing and tracking developmental change in longitudinal research requires a reliable measure with appropriate measurement invariance properties. Put otherwise, it is important to establish whether or not the SWEMWBS means the same thing as young people experience a period of rapid developmental change. It may be that individual items take on different meanings as young people grow up, and young people’s experiences may relate more or less strongly to different items of the questionnaire.

Previous research examining this question is limited. A study of SWEMWBS in a sample of 829 Australian adolescents aged 13–16 found acceptable measurement invariance properties by age, but examined a restricted number of age groups from seven secondary schools [13]. In addition, it is important to consider whether the SWEMWBS has good external construct validity in population, rather than selective or interventional, samples of young people. This is a question heretofore not investigated in the literature.

## Methods

Our aim was to use a ‘real-world’ population sample (*n* > 94,000) of young people in Wales to examine the psychometric properties of SWEMWBS in young people aged 11–16. Specifically, we sought to a) examine the measurement invariance properties of SWEMWBS across the full age range of secondary school students, and b) consider the external construct validity of SWEMWBS using other indicators (life satisfaction, somatisation, school pressure and bullying victimisation) relevant to policy and practice.

### Study sample

We used data from the 2017 School Health Research Network Student Health and Wellbeing Survey [14], completed by 103,971 students in years 7 to 11 from 193 secondary schools in Wales. Schools were recruited to the Network either via participation in the Welsh Health Behaviour in School-aged Children (HBSC) survey in 2013–2014, or via two rounds of open recruitment. All Network member schools (*n* = 212) were invited to take part in the survey. The Student Health and Wellbeing Survey is an online, closed response, self-completion survey, available in English and Welsh. The survey measures self-reported health behaviours among school students aged 11–16 years (i.e. in years 7 to 11 of the British secondary school system), and includes questions from the current round of the international HBSC survey [15] alongside additional questions reflecting current policy, practice and research priorities in Wales. Students completed the survey during school hours between September and December of the autumn term of the 2017–2018 school year. Students could opt out of the survey.

### Measurement invariance properties of SWEMWBS

All students were presented with the seven questions comprising SWEMWBS: ‘I’ve been feeling optimistic about the future’, ‘I’ve been feeling useful’, ‘I’ve been feeling relaxed’, ‘I’ve been dealing with problems well’, ‘I’ve been thinking clearly’, ‘I’ve been feeling close to other people’, and ‘I’ve been able to make up my own mind about things’ alongside with a question stem: ‘Below are some statements about feelings and thoughts. Please select the option that best describes your experience of each over the last 2 weeks’. For each question, students could select one of five frequency options: ‘none of the time’, ‘rarely’, ‘some of the time’, ‘often’ and ‘all of the time’. Observations where students either responded ‘I do not want to answer’ or left a question blank were set to missing.

We first estimated polychoric correlation matrices for the whole sample and by school year, and undertook a principal components analysis to check for configural invariance (i.e. is the number of factors equal for each year group?). We used a parallel analysis routine with 10,000 draws to verify the number of factors to be extracted overall and in each year group. Subsequently, we used a multiple-groups structural equation model with successively greater constraints to test measurement invariance. Because of the ordinal nature of the individual items, we used a diagonally weighted least squares estimator with a scale-shifted test statistic. Briefly, this estimator treats ordinal variables as reflecting an underlying response variable by ‘mapping’ thresholds between each value of the ordinal variable onto a normal distribution, and generates robust sandwich standard errors for model parameters [16]. These thresholds take the place of intercepts normally seen with continuous indicators.

In keeping with standard measurement invariance testing procedures, our first model assumed only configural invariance. We used this model to examine item functioning over the different year groups. Our second model restricted loadings to be equal across groups. Our third model additionally restricted thresholds for indicators to be equal across groups. Our fourth and final model set residual variance for each indicator equal across groups. Because of the size of our sample and hence the risk of spurious significance, we did not use traditional χ^2^ tests for invariance. Instead, we used the comparative fit index (CFI) and the root mean squared error of approximation (RMSEA). The CFI has previously been shown to be an appropriate index of measurement invariance, with decrements of greater than − 0.01 in successive models suggesting that measurement variance is not appropriate [17]. In addition, emerging evidence shows promise for the RMSEA as an information criterion, where the lowest value indicates the model with the best trade-off between fit and complexity [18]. For the best-fitting model, we benchmarked the RMSEA against a criterion of 0.05 for acceptable fit, alongside a one-sided hypothesis test of equality against this criterion (i.e. *p*-value of close fit; [19]. Both indices were calculated using the scale-shifted χ^2^ test statistic. Given the sample size used in this analysis and the risks of multiple testing, we did not test partial invariance models. As a robustness check, we repeated our measurement invariance tests using a maximum likelihood estimator with robust standard errors.

### External construct validity of SWEMWBS

Based on theory [1, 20, 21], evidence and available data, we selected four covariates to examine the external construct validity of SWEMWBS. We included two measures of related constructs: life satisfaction and somatisation. Life satisfaction was measured using Cantril’s self-anchoring ladder [22], with a score of 0 indicating ‘the worst possible life’ and a score of 10 indicating ‘the best possible life’. Somatisation included frequency in the last 6 months of: feeling low, irritability or bad temper, feeling nervous, or difficulties in getting to sleep, where students could respond ‘about every day’, ‘more than once a week’, ‘about every week’, ‘about every month’, or ‘rarely or never’. Scores for each of the four symptoms were summed to create a scale (range 5–20). Items for somatisation were part of the core set of questions for the HBSC survey. We also included two measures of well-established risk factors for poor mental health and wellbeing: school pressure [23] and bullying victimisation [24]. Students were asked ‘How pressured do you feel by the schoolwork you have to do?’ with response options ‘not at all’, ‘a little’, ‘some’, ‘a lot’. Students were also asked ‘How often have you been bullied at school in the past couple of months?’ with response options ‘I have not been bullied at school in the past couple of months’, ‘it has happened once or twice’, ‘2 or 3 times a month’, ‘about once a week’, or ‘several times a week’. For all covariates, observations where students either responded ‘I do not want to answer’ or left a question blank were set to missing.

To examine external construct validity, we calculated correlations using Pearson’s *r* or Spearman’s rho as appropriate between the SWEMWBS scale score and each of the covariates. We estimated correlations both across all year groups and for each year group separately.

We undertook analyses using lavaan [25] in the R computing environment and Stata v.14 (Statacorp, College Station, TX).

## Results

From the whole sample of 103,971 respondents (73% response rate compared to all students in participating schools), our sample included 94,476 adolescents with responses to all seven SWEMWBS questions (91% of the respondents, or 66% of the sampling population). Descriptive statistics for the sample, including item-level frequencies for SWEMWBS questions and key covariates, can be found in Table 1. Within the analysis sample, most covariates had low missingness (2% for life satisfaction, 5% for both school pressure and somatisation, 13% for bullying victimisation).

**Table 1:** Sample and item descriptive statistics

Variable	Mean (SD)	Response options N (%)				
*Gender*						
Male		45,961 (49)				
Female		46,977 (50)				
Prefer not to say		1538 (2)				
*School year*						
Year 7		19,532 (21)				
Year 8		19,960 (21)				
Year 9		20,354 (22)				
Year 10		18,415 (19)				
Year 11		16,215 (17)				
*Family affluence*						
Low		30,733 (34)				
Medium		28,363 (31)				
High		31,252 (35)				
*Ethnicity*						
White British		79,380 (86)				
White non-British		4302 (5)				
BME		8546 (9)				
*SWEMWBS items*		None of the time	Rarely	Some of the time	Often	All of the time
*Item 1* “I’ve been feeling optimistic about the future”		8936 (9)	17,633 (19)	28,578 (30)	26,825 (28)	12,504 (13)
*Item 2* “I’ve been feeling useful”		7070 (7)	17,073 (18)	32,657 (35)	28,954 (31)	8722 (9)
*Item 3* “I’ve been feeling relaxed”		5630 (6)	16,115 (17)	26,771 (28)	31,934 (34)	14,026 (15)
*Item 4* “I’ve been dealing with problems well”		7636 (8)	15,414 (16)	28,701 (30)	29,655 (31)	13,070 (14)
*Item 5* “I’ve been thinking clearly”		5020 (5)	13,017 (14)	26,759 (28)	32,610 (35)	17,070 (18)
*Item 6* “I’ve been feeling close to other people”		5109 (5)	10,989 (12)	21,543 (23)	31,032 (33)	25,803 (27)
*Item 7* “I’ve been able to make up my own mind about things”		3396 (4)	7799 (8)	17,972 (19)	32,901 (35)	32,408 (34)
*School pressure*		Not at all	A little	Some	A lot	
		15,179 (17)	31,292 (35)	21,613 (24)	21,697 (24)	
*Been bullied*		Haven’t	Once or twice	2 or 3 times a month	About once a week	Several times a week
		53,203 (64)	17,558 (21)	4326 (5)	3011 (4)	4561 (6)
*Somatising symptomatology*		Rarely or never	About every month	About every week	More than once a week	About everyday
Feeling low		38,074 (41	17,934 (19)	13,484 (15)	13,263 (14)	9348 (10)
Irritable		29,876 (32)	18,502 (20)	15,744 (17)	15,825 (17)	12,417 (13)
Nervous		33,964 (37)	20,447 (22)	15,041 (16)	12,403 (13)	10,929 (12)
Sleep difficulties		42,078 (45)	12,050 (13)	9908 (11)	12,363 (13)	16,392 (18)
*Life satisfaction*	7.51 (11.66)					

### Configural invariance

Parallel analysis suggested that extraction of one factor was appropriate both overall and in each year group. This was reflected by the principal components analysis, which indicated that one component explained 51.7% of the variance in the whole sample. The proportion of variance explained by one factor ranged from 48.6% in year 7 students to 54.8% in year 11 students, with this proportion increasing by year. Visual examination of polychoric correlation matrices (see Additional file 1: Table S1) did not reveal obvious differences in item intercorrelations by year group.

### Measurement invariance

Having verified unidimensionality of the SWEMWBS in each year group, we estimated a one-factor configural invariance model. Parameter estimates from this model are shown in Table 2. (Threshold estimates are omitted for clarity and are presented in Additional file 1: Table S2.) Inspection of standardised loadings suggested that four items, specifically questions 1, 2, 4 and 5, had progressively stronger correlations with the factor as students are older. This was mirrored by a decrease in residual variance for each of these items over year group.

**Table 2:** Parameter estimates for overall and configural invariance models

Group	Unstandardised loading	SE	*z*-score	Standardised loading	Variance
All years					
Q1	1			0.51	0.74
Q2	1.34	0.007	188.569	0.68	0.54
Q3	1.33	0.007	182.57	0.67	0.55
Q4	1.39	0.007	186.03	0.70	0.50
Q5	1.56	0.008	191.564	0.79	0.38
Q6	1.19	0.007	173.104	0.60	0.64
Q7	1.32	0.007	179.486	0.67	0.55
Year 7					
Q1	1			0.48	0.78
Q2	1.35	0.02	72.32	0.64	0.59
Q3	1.36	0.02	70.64	0.65	0.58
Q4	1.39	0.02	71.30	0.66	0.57
Q5	1.57	0.02	74.12	0.75	0.44
Q6	1.25	0.02	69.23	0.59	0.65
Q7	1.41	0.02	70.65	0.67	0.55
Year 8					
Q1	1			0.46	0.79
Q2	1.39	0.02	71.79	0.65	0.58
Q3	1.44	0.02	71.42	0.67	0.55
Q4	1.47	0.02	71.89	0.68	0.54
Q5	1.65	0.02	73.53	0.77	0.41
Q6	1.27	0.02	67.48	0.59	0.65
Q7	1.41	0.02	70.05	0.65	0.57
Year 9					
Q1	1			0.51	0.74
Q2	1.37	0.02	87.39	0.69	0.52
Q3	1.34	0.02	84.94	0.68	0.54
Q4	1.38	0.02	85.91	0.70	0.51
Q5	1.56	0.02	88.73	0.79	0.37
Q6	1.19	0.02	80.36	0.60	0.64
Q7	1.31	0.02	82.63	0.67	0.56
Year 10					
Q1	1			0.56	0.68
Q2	1.26	0.01	101.14	0.71	0.49
Q3	1.18	0.01	94.60	0.67	0.56
Q4	1.32	0.01	100.04	0.74	0.45
Q5	1.43	0.01	103.19	0.80	0.35
Q6	1.07	0.01	88.22	0.61	0.63
Q7	1.19	0.01	92.93	0.67	0.55
Year 11					
Q1	1			0.59	0.66
Q2	1.23	0.01	101.44	0.72	0.48
Q3	1.15	0.01	94.82	0.68	0.55
Q4	1.29	0.01	99.84	0.76	0.43
Q5	1.39	0.01	103.62	0.81	0.34
Q6	1.03	0.01	86.13	0.61	0.63
Q7	1.15	0.01	92.09	0.67	0.55

Results from each of the successively stricter invariance tests are reported in Table 3. As expected, each χ^2^ test suggested significantly worse fit with increasing model constraints. However, both the CFI and the RMSEA suggested that a model with loadings and thresholds constrained to be equal across year groups was satisfactory. The decrement between the loadings-only model and the ‘loadings and thresholds’ model was less than 0.01 in the CFI, while a model that also constrained residual variances to be equal across groups showed a decrement in fit greater than 0.01, indicating that measurement invariance was not supported in this more restrictive model. Similarly, the RMSEA was lowest for the ‘loadings and thresholds’ model, indicating that this was the preferred model. In absolute terms, the RMSEA for the loadings and thresholds model suggested ‘good’ model fit, with a 90% confidence interval of 0.050 to 0.051 and a *p*-value of close fit of 0.174.

**Table 3:** Measurement invariance tests

Model constraints	CFI	RMSEA	Degrees of freedom	χ2	χ2 difference	*p*-value
Configural	0.979	0.078	70	3808.5		
Loadings	0.983	0.060	94	4014.0	204.1	< 0.001
Loadings, thresholds	0.978	0.051	174	6049.5	2480.6	< 0.001
Loadings, thresholds, residuals	0.958	0.064	202	11,812.5	6169.4	< 0.001

### External construct validity

SWEMWBS scores were moderately correlated with measures of life satisfaction and somatisation (see Table 4). Of note is that correlation between the SWEMWBS score and each covariate increased by year group. SWEMWBS score was also correlated with school pressure and bullying victimisation, though relationships were weak to moderate and did not show an increasing trend with age. All correlations were statistically significant at the *p* < 0.001 level.

**Table 4:** Correlations between SWEMWBS and key covariates

Year Group	Life satisfaction	Somatisation	School pressure	Bullying victimisation
All years	0.50	−0.53	−0.30	−0.30
Year 7	0.43	−0.45	−0.29	−0.25
Year 8	0.48	−0.51	−0.30	−0.32
Year 9	0.50	−0.54	−0.27	−0.33
Year 10	0.51	−0.54	−0.25	−0.30
Year 11	0.52	−0.57	−0.28	−0.31

### Robustness analysis

Measurement invariance testing undertaken with a maximum likelihood estimator with robust errors also suggested that a loadings and thresholds model was acceptable (see Additional file 1: Table S3).

## Discussion

In this analysis, we show for the first time, and using a national sample, that the SWEMWBS has satisfactory measurement invariance properties in secondary school students in years 7 to 11 (ages 11 to 16). Moreover, a model with equal loadings and thresholds across age groups had ‘good’ fit, with a 90% confidence interval for the RMSEA of 0.050 to 0.051 and a *p*-value of close fit of 0.174. This extends the utility of the SWEMWBS to an age group where only the questionnaire’s longer form had previously been validated, for example, a recent study showed that better teacher wellbeing was associated with better student wellbeing and lower student psychological distress in a sample of 3000+ year 8 students in England and Wales [26]. We also provide initial evidence that the SWEMWBS has satisfactory external construct validity in this age group.

Our findings regarding measurement invariance specifically suggest that age differences in SWEMWBS can be attributed to developmental differences in the underlying latent trait rather than to the measure itself. However, our finding regarding the relatively poorer fit of the ‘loadings, thresholds and residuals constrained’ model suggests that SWEMWBS will measure its underlying construct, mental wellbeing, with decreasing measurement error in older ages. This relationship between age and measurement error was borne out in our initial finding that one component explained an increasing proportion of variance with increasing year group, and later in our tests of external construct validity with respect to related constructs. In addition, our finding that questions 1, 2, 4 and 5 had progressively stronger correlations with older age groups suggests that these questions may have increasing salience and relevance throughout adolescence. It was likely that these questions drove the decreases in measurement error in older age groups, and may reflect particular aspects of mental wellbeing that develop most strongly over the age range considered in this study.

Given the length constraints of the Student Health and Wellbeing survey, we had access to a limited number of relevant covariates in testing external construct validity. Our demonstration of moderate to strong relationships between SWEMWBS and each of life satisfaction and somatisation adds additional evidence regarding external construct validity. A prior validation study of the full WEMWBS scale in young people aged 13 to 16 used the World Health Organization WHO-5 Wellbeing Index, Kidscreen-27, Mental Health Continuum-Short Form, the General Health Questionnaire and the Strengths and Difficulties Questionnaire [7]. While each of these questionnaires is appropriate to measure mental health and wellbeing, the use of all of these in a population survey context would be challenging. We showed moderate relationships between SWEMWBS and two short, practical measures of mental health and wellbeing, providing ‘real-world’ evidence of the external construct validity of SWEMWBS in respect of other commonly used and internationally relevant measures. In the original validation study of the full WEMWBS in young people [7], correlations with the scales used to test external construct validity ranged in magnitude from 0.38 to 0.65. Our results compare favourably with these estimates. Moreover, our decision to use bullying victimisation and school pressure as risk factors were informed by policy priorities and epidemiological evidence. Our findings for relationships between these risk factors and SWEMWBS add yet more evidence for the external construct validity of SWEMWBS in population survey settings. However, it remains an open question the degree to which mental wellbeing is an empirically different construct than mental illness. Evidence from the Health Survey for England suggests that WEMWBS measures the same construct as the GHQ-12, a general measure of psychological distress [25]; yet at the population level, youth mental distress has increased [27] at the same time as youth wellbeing has improved [28], and correlates of mental wellbeing do not overlap comprehensively with correlates of mental ill health [29]. In other words, is mental wellbeing a ‘positively worded’ version of mental illness symptomatology questions? If this is the case, then one possible merit of SWEMWBS might be greater acceptability and hence higher completion rates, especially in young people.

To our knowledge, most major measurement validation studies of SWEMWBS have been undertaken in adults. Haver and colleagues [8] showed acceptable construct validity, using mindfulness, emotional intelligence, and positive and negative affect in a sample of Scandinavian hotel managers. Smith and colleagues [9] used depression, generalised anxiety disorder, functional health, mindfulness and self-control to test external construct validity in a sample of Norwegian patients seeking treatment for mental health conditions. Though both studies showed acceptable external construct validity of SWEMWBS, Smith and colleagues [9] also showed that in their adult sample, SWEMWBS only attained satisfactory measurement invariance with correlated residual errors. We did not correlate residual errors as this would have complicated interpretation and calculation of the scale score and was not necessary to demonstrate good model fit. We note that the question of correlated errors has been raised by multiple studies that have found that this has increased model fit [9, 13]; while it was beyond the scope of this particular study, it would be of scientific interest to consider what gives rise to these correlated errors, and how these error correlations might change over the age range. However, similar to Smith, we found that a loadings and thresholds model yielded good model fit. Finally, Hunter, Houghton and Wood [13] showed in a sample of 829 Australian adolescents that a loadings and thresholds model for the SWEMWBS had acceptable measurement invariance properties, but their sample was restricted to ages 13 to 16. We extend these findings to younger ages and provide evidence of external construct validity in adolescents.

Our analysis has several strengths, but also several limitations. First, we used a large-scale population sample to undertake the largest validation of WEMWBS to date. Our nationally representative sample provides yet more evidence of the utility of SWEMWBS for measuring mental wellbeing among young people in the United Kingdom. Second, we were able to use other public health-relevant questions to examine external construct validity of SWEMWBS in adolescents. However, unlike the original validation studies for WEMWBS and SWEMWBS, we were unable to consider external construct validity using clinical measures of mental ill health and other measures designed to assess mental wellbeing. We were also unable to consider test-retest reliability. Additionally, it is possible that results from Wales and the United Kingdom may not generalise internationally, though evidence of the psychometric properties of SWEMWBS in adults is consistent across multiple cultures [30]. Future research should seek to test the external construct validity of SWEMWBS in population samples using alternative wellbeing measures and a wider range of independently assessed criteria (e.g. self-harm, educational success), consider the questionnaire’s utility in primary school populations, and understand any potential benefits of SWEMWBS over similar general measures of mental wellbeing or psychological distress.

## Conclusions

This study adds to the growing evidence that SWEMWBS is appropriate, if not better than the full WEMWBS [5, 13], for measuring mental wellbeing in young people, provides evidence for the utility of SWEMWBS in younger age groups than before, and suggests that SWEMWBS is appropriate for tracking the development of wellbeing across adolescence.

## Additional file

Additional file 1: **Table S1.** Overall and year-specific polychoric correlation matrices. **Table S2.** Threshold estimates for overall and configural invariance models. **Table S3.** Measurement invariance tests using maximum likelihood estimation with robust standard errors. (DOCX 36 kb)

## Data Availability

Due to the sensitive nature of the questions asked in this study, survey respondents were assured raw data would remain confidential and would not be shared.

## Abbreviations

CFI: comparative fit index
DECIPHer: The Centre for the Development and Evaluation of Complex Interventions for Public Health Improvement
GHQ: General Health Questionnaire
HBSC: Health Behaviour in School-aged Children
RMSEA: Root mean squared error of approximation
SWEMWBS: Short Warwick-Edinburgh Mental Wellbeing Scale
UKCRC: UK Clinical Research Collaboration
WEMWBS: Warwick-Edinburgh Mental Wellbeing Scale
WHO: World Health Organisation
