# Supplementary material for: Measurement invariance properties and external construct validity of the short Warwick-Edinburgh mental wellbeing scale in a large national sample of secondary school students in Wales
Source: Health Qual Life Outcomes. 2019 Aug 14;17:139. doi: 10.1186/s12955-019-1204-z (PMC6694652; doi:10.1186/s12955-019-1204-z)
Supplement: Supplementary file 1 — Table S1. Overall and year-specific polychoric correlation matrices. Table S2. Threshold estimates for overall and configural invariance models. Table S3. Measurement invariance tests using maximum likelihood estimation with robust standard errors. (DOCX 36 kb) [file 12955_2019_1204_MOESM1_ESM.docx]

**Additional file**

**Table S1: Overall and year-specific polychoric correlation matrices.**

| **Year group** | | | | | | | |
| --- | --- | --- | --- | --- | --- | --- | --- |
| All years | | | | | | | |
|  | Q1 | Q2 | Q3 | Q4 | Q5 | Q6 | Q7 |
| Q1 | 1.00 |  |  |  |  |  |  |
| Q2 | 0.45 | 1.00 |  |  |  |  |  |
| Q3 | 0.29 | 0.47 | 1.00 |  |  |  |  |
| Q4 | 0.33 | 0.46 | 0.47 | 1.00 |  |  |  |
| Q5 | 0.35 | 0.50 | 0.54 | 0.58 | 1.00 |  |  |
| Q6 | 0.32 | 0.42 | 0.41 | 0.40 | 0.45 | 1.00 |  |
| Q7 | 0.32 | 0.42 | 0.44 | 0.47 | 0.55 | 0.43 | 1.00 |
| Year 7 | | | | | | | |
|  | Q1 | Q2 | Q3 | Q4 | Q5 | Q6 | Q7 |
| Q1 | 1.00 |  |  |  |  |  |  |
| Q2 | 0.39 | 1.00 |  |  |  |  |  |
| Q3 | 0.26 | 0.42 | 1.00 |  |  |  |  |
| Q4 | 0.30 | 0.41 | 0.42 | 1.00 |  |  |  |
| Q5 | 0.31 | 0.44 | 0.50 | 0.52 | 1.00 |  |  |
| Q6 | 0.31 | 0.39 | 0.37 | 0.37 | 0.42 | 1.00 |  |
| Q7 | 0.29 | 0.39 | 0.43 | 0.43 | 0.53 | 0.41 | 1.00 |
| Year 8 | | | | | | | |
|  | Q1 | Q2 | Q3 | Q4 | Q5 | Q6 | Q7 |
| Q1 | 1.00 |  |  |  |  |  |  |
| Q2 | 0.39 | 1.00 |  |  |  |  |  |
| Q3 | 0.27 | 0.44 | 1.00 |  |  |  |  |
| Q4 | 0.30 | 0.43 | 0.45 | 1.00 |  |  |  |
| Q5 | 0.31 | 0.46 | 0.53 | 0.55 | 1.00 |  |  |
| Q6 | 0.30 | 0.39 | 0.40 | 0.39 | 0.42 | 1.00 |  |
| Q7 | 0.27 | 0.39 | 0.42 | 0.44 | 0.53 | 0.41 | 1.00 |
| Year 9 | | | | | | | |
|  | Q1 | Q2 | Q3 | Q4 | Q5 | Q6 | Q7 |
| Q1 | 1.00 |  |  |  |  |  |  |
| Q2 | 0.45 | 1.00 |  |  |  |  |  |
| Q3 | 0.29 | 0.49 | 1.00 |  |  |  |  |
| Q4 | 0.33 | 0.47 | 0.47 | 1.00 |  |  |  |
| Q5 | 0.36 | 0.51 | 0.55 | 0.58 | 1.00 |  |  |
| Q6 | 0.31 | 0.42 | 0.41 | 0.40 | 0.46 | 1.00 |  |
| Q7 | 0.32 | 0.42 | 0.44 | 0.46 | 0.55 | 0.44 | 1.00 |
| Year 10 | | | | | | | |
|  | Q1 | Q2 | Q3 | Q4 | Q5 | Q6 | Q7 |
| Q1 | 1.00 |  |  |  |  |  |  |
| Q2 | 0.51 | 1.00 |  |  |  |  |  |
| Q3 | 0.32 | 0.48 | 1.00 |  |  |  |  |
| Q4 | 0.38 | 0.51 | 0.50 | 1.00 |  |  |  |
| Q5 | 0.41 | 0.54 | 0.55 | 0.63 | 1.00 |  |  |
| Q6 | 0.35 | 0.44 | 0.41 | 0.42 | 0.47 | 1.00 |  |
| Q7 | 0.36 | 0.44 | 0.44 | 0.49 | 0.56 | 0.44 | 1.00 |
| Year 11 | | | | | | | |
|  | Q1 | Q2 | Q3 | Q4 | Q5 | Q6 | Q7 |
| Q1 | 1.00 |  |  |  |  |  |  |
| Q2 | 0.54 | 1.00 |  |  |  |  |  |
| Q3 | 0.34 | 0.49 | 1.00 |  |  |  |  |
| Q4 | 0.39 | 0.51 | 0.53 | 1.00 |  |  |  |
| Q5 | 0.43 | 0.55 | 0.56 | 0.65 | 1.00 |  |  |
| Q6 | 0.34 | 0.45 | 0.42 | 0.43 | 0.47 | 1.00 |  |
| Q7 | 0.38 | 0.45 | 0.44 | 0.50 | 0.56 | 0.45 | 1.00 |

**Table S2: Threshold estimates for overall and configural invariance models.**

| **Question\|**  **threshold** | **Overall** | | | **Year 7** | | | **Year 8** | | | **Year 9** | | | **Year 10** | | | **Year 11** | | |
| --- | --- | --- | --- | --- | --- | --- | --- | --- | --- | --- | --- | --- | --- | --- | --- | --- | --- | --- |
|  | **Estimate** | **SE** | **z-value** | **Estimate** | **SE** | **z-value** | **Estimate** | **SE** | **z-value** | **Estimate** | **SE** | **z-value** | **Estimate** | **SE** | **z-value** | **Estimate** | **SE** | **z-value** |
| Q1\|t1 | -1.31 | 0.01 | -232.35 | -1.16 | 0.01 | -100.39 | -1.27 | 0.01 | -105.44 | -1.35 | 0.01 | -108.71 | -1.38 | 0.01 | -104.07 | -1.48 | 0.02 | -98.93 |
| Q1\|t2 | -0.58 | 0.004 | -133.58 | -0.53 | 0.01 | -55.71 | -0.55 | 0.01 | -58.17 | -0.59 | 0.01 | -62.64 | -0.59 | 0.01 | -59.89 | -0.67 | 0.01 | -62.66 |
| Q1\|t3 | 0.21 | 0.004 | 51.43 | 0.13 | 0.01 | 14.58 | 0.20 | 0.01 | 22.79 | 0.24 | 0.01 | 27.11 | 0.27 | 0.01 | 28.54 | 0.22 | 0.01 | 22.04 |
| Q1\|t4 | 1.12 | 0.01 | 216.68 | 0.94 | 0.01 | 89.23 | 1.10 | 0.01 | 98.74 | 1.18 | 0.01 | 103.21 | 1.21 | 0.01 | 99.36 | 1.20 | 0.01 | 92.80 |
| Q2\|t1 | -1.44 | 0.01 | -237.83 | -1.40 | 0.01 | -107.49 | -1.44 | 0.01 | -109.36 | -1.45 | 0.01 | -110.54 | -1.43 | 0.01 | -104.84 | -1.49 | 0.02 | -99.01 |
| Q2\|t2 | -0.66 | 0.004 | -148.87 | -0.75 | 0.01 | -74.98 | -0.71 | 0.01 | -73.19 | -0.63 | 0.01 | -66.88 | -0.59 | 0.01 | -60.23 | -0.59 | 0.01 | -56.55 |
| Q2\|t3 | 0.26 | 0.004 | 62.15 | 0.06 | 0.01 | 6.77 | 0.17 | 0.01 | 19.50 | 0.30 | 0.01 | 33.34 | 0.39 | 0.01 | 40.59 | 0.41 | 0.01 | 40.21 |
| Q2\|t4 | 1.33 | 0.01 | 233.10 | 1.10 | 0.01 | 97.88 | 1.26 | 0.01 | 105.25 | 1.39 | 0.01 | 109.50 | 1.49 | 0.01 | 105.47 | 1.52 | 0.02 | 99.17 |
| Q3\|t1 | -1.56 | 0.01 | -239.72 | -1.58 | 0.01 | -109.01 | -1.62 | 0.02 | -110.12 | -1.59 | 0.01 | -111.27 | -1.51 | 0.01 | -105.64 | -1.49 | 0.02 | -99.01 |
| Q3\|t2 | -0.74 | 0.01 | -163.77 | -0.88 | 0.01 | -84.86 | -0.82 | 0.01 | -81.29 | -0.77 | 0.01 | -78.45 | -0.64 | 0.01 | -63.97 | -0.58 | 0.01 | -55.28 |
| Q3\|t3 | 0.03 | 0.004 | 8.32 | -0.16 | 0.01 | -17.50 | -0.05 | 0.01 | -6.02 | 0.02 | 0.01 | 2.52 | 0.17 | 0.01 | 17.92 | 0.24 | 0.01 | 24.00 |
| Q3\|t4 | 1.04 | 0.01 | 208.79 | 0.83 | 0.01 | 81.75 | 0.99 | 0.01 | 92.72 | 1.09 | 0.01 | 99.25 | 1.19 | 0.01 | 98.79 | 1.20 | 0.01 | 92.89 |
| Q4\|t1 | -1.40 | 0.01 | -236.46 | -1.38 | 0.01 | -107.06 | -1.42 | 0.01 | -108.94 | -1.39 | 0.01 | -109.62 | -1.38 | 0.01 | -104.00 | -1.45 | 0.02 | -98.60 |
| Q4\|t2 | -0.69 | 0.004 | -155.69 | -0.76 | 0.01 | -75.99 | -0.72 | 0.01 | -73.62 | -0.69 | 0.01 | -71.98 | -0.64 | 0.01 | -64.02 | -0.66 | 0.01 | -61.76 |
| Q4\|t3 | 0.12 | 0.004 | 29.36 | -0.03 | 0.01 | -3.26 | 0.07 | 0.01 | 8.32 | 0.13 | 0.01 | 15.21 | 0.22 | 0.01 | 23.57 | 0.23 | 0.01 | 23.03 |
| Q4\|t4 | 1.09 | 0.01 | 213.81 | 0.88 | 0.01 | 84.71 | 1.02 | 0.01 | 94.40 | 1.14 | 0.01 | 101.73 | 1.26 | 0.01 | 101.01 | 1.24 | 0.01 | 94.28 |
| Q5\|t1 | -1.62 | 0.01 | -239.58 | -1.73 | 0.02 | -107.96 | -1.65 | 0.02 | -109.90 | -1.60 | 0.01 | -111.25 | -1.54 | 0.02 | -105.79 | -1.56 | 0.02 | -99.31 |
| Q5\|t2 | -0.88 | 0.01 | -186.14 | -1.10 | 0.01 | -97.66 | -0.95 | 0.01 | -90.32 | -0.85 | 0.01 | -84.86 | -0.75 | 0.01 | -73.03 | -0.74 | 0.01 | -67.59 |
| Q5\|t3 | -0.07 | 0.004 | -15.89 | -0.36 | 0.01 | -39.47 | -0.15 | 0.01 | -17.26 | -0.03 | 0.01 | -3.80 | 0.11 | 0.01 | 11.74 | 0.16 | 0.01 | 15.96 |
| Q5\|t4 | 0.91 | 0.01 | 191.79 | 0.58 | 0.01 | 61.15 | 0.83 | 0.01 | 82.49 | 0.97 | 0.01 | 92.96 | 1.14 | 0.01 | 96.69 | 1.20 | 0.01 | 93.08 |
| Q6\|t1 | -1.61 | 0.01 | -239.65 | -1.53 | 0.01 | -108.89 | -1.65 | 0.02 | -109.92 | -1.63 | 0.02 | -111.12 | -1.59 | 0.02 | -105.83 | -1.64 | 0.02 | -99.13 |
| Q6\|t2 | -0.95 | 0.01 | -197.36 | -0.97 | 0.01 | -90.53 | -1.00 | 0.01 | -93.35 | -0.98 | 0.01 | -92.99 | -0.91 | 0.01 | -84.64 | -0.91 | 0.01 | -78.96 |
| Q6\|t3 | -0.26 | 0.004 | -62.38 | -0.35 | 0.01 | -37.85 | -0.34 | 0.01 | -37.44 | -0.26 | 0.01 | -29.34 | -0.17 | 0.01 | -18.55 | -0.15 | 0.01 | -14.80 |
| Q6\|t4 | 0.60 | 0.004 | 138.42 | 0.42 | 0.01 | 45.29 | 0.52 | 0.01 | 55.46 | 0.63 | 0.01 | 66.65 | 0.73 | 0.01 | 71.87 | 0.78 | 0.01 | 70.99 |
| Q7\|t1 | -1.80 | 0.01 | -234.70 | -1.79 | 0.02 | -106.88 | -1.86 | 0.02 | -106.63 | -1.79 | 0.02 | -109.20 | -1.76 | 0.02 | -104.29 | -1.81 | 0.02 | -97.12 |
| Q7\|t2 | -1.18 | 0.01 | -222.98 | -1.27 | 0.01 | -104.32 | -1.21 | 0.01 | -103.41 | -1.18 | 0.01 | -103.23 | -1.14 | 0.01 | -96.55 | -1.12 | 0.01 | -90.07 |
| Q7\|t3 | -0.50 | 0.004 | -117.03 | -0.64 | 0.01 | -66.35 | -0.56 | 0.01 | -59.58 | -0.50 | 0.01 | -53.83 | -0.42 | 0.01 | -43.54 | -0.37 | 0.01 | -36.61 |
| Q7\|t4 | 0.40 | 0.004 | 96.22 | 0.24 | 0.01 | 26.68 | 0.34 | 0.01 | 37.51 | 0.40 | 0.01 | 43.92 | 0.52 | 0.01 | 53.88 | 0.57 | 0.01 | 54.48 |

**Table S3: Measurement invariance tests using maximum likelihood estimation with robust standard errors.**

| **Model constraints** | **CFI** | **RMSEA** | **Degrees of freedom** | **χ2** | **χ2 difference** | ***p*-value** |
| --- | --- | --- | --- | --- | --- | --- |
| Configural | 0.972 | 0.053 | 70 | 4930.6 |  |  |
| Loadings | 0.970 | 0.048 | 94 | 5042.4 | 110.9 | <0.001 |
| Loadings, thresholds | 0.960 | 0.049 | 118 | 6518.7 | 1474.8 | <0.001 |
| Loadings, thresholds, residuals | 0.938 | 0.055 | 146 | 9938.8 | 2948.7 | <0.001 |
